# Differential Treatment of Older Workers Due to COVID-19: Potential for Ageism and Age Discrimination at Work

**DOI:** 10.1093/geroni/igab046.1711

**Published:** 2021-12-17

**Authors:** Lisa Hollis-Sawyer

**Affiliations:** Northeastern Illinois University, Chicago, Illinois, United States

## Abstract

This paper examines the implications of employers' current COVID-19 protective workplace attendance policies toward older workers, potentially creating the outcomes of increased numbers of involuntary retirees and the discouraged older worker syndrome among otherwise qualified older workforce participants. How potential ageist assumptions and age discrimination under COVID-19 affect workplace decisions in reflection on the Age Discrimination in Employment Act (1967) guidelines is discussed. Older workers may remain in the workforce longer than ever before due to having healthier life expectancies. Workplace policies need to be increasingly sensitive to older employees’ rights to sustain their workplace engagement (Cummins, 2014; Cummins, Harootyan, & Kunkel, 2015). The author reviewed current unemployment trends in 2020 and emerging litigation in reflection upon general issues of COVID-19 related age discrimination in the older workers' workplace attendance decisions by employers and the historical framework of the Age Discrimination in Employment Act (1967, with significant amendments in 1978 and 1986). The policy analysis paper presents the implications of employers' COVID-19 protective policies on older workers and how it may affect the “health” of the workplace and older adults and the economy beyond the pandemic. Lastly, strategies to address an "age-friendly" workplace during a pandemic and post-pandemic are discussed.

